# Task load modulates tDCS effects on brain network for phonological processing

**DOI:** 10.1007/s10339-020-00964-w

**Published:** 2020-03-10

**Authors:** Lílian Rodrigues de Almeida, Paul A. Pope, Peter C. Hansen

**Affiliations:** School of Psychology, University of Birmingham, Edgbaston, Birmingham, B15 2TT UK

**Keywords:** Phonological processing, Task load, LIFG, Language, fMRI, tDCS

## Abstract

Motor participation in phonological processing can be modulated by task nature across the speech perception to speech production range. The pars opercularis of the left inferior frontal gyrus (LIFG) would be increasingly active across this range, because of changing motor demands. Here, we investigated with simultaneous tDCS and fMRI whether the task load modulation of tDCS effects translates into predictable patterns of functional connectivity. Findings were analysed under the “multi-node framework”, according to which task load and the network structure underlying cognitive functions are modulators of tDCS effects. In a within-subject study, participants (*N* = 20) performed categorical perception, lexical decision and word naming tasks [which differentially recruit the target of stimulation (LIFG)], which were repeatedly administered in three tDCS sessions (anodal, cathodal and sham). The LIFG, left superior temporal gyrus and their right homologues formed the target network subserving phonological processing. C-tDCS inhibition and A-tDCS excitation should increase with task load. Correspondingly, the larger the task load, the larger the relevance of the target for the task and smaller the room for compensation of C-tDCS inhibition by less relevant nodes. Functional connectivity analyses were performed with partial correlations, and network compensation globally inferred by comparing the relative number of significant connections each condition induced relative to sham. Overall, simultaneous tDCS and fMRI was adequate to show that motor participation in phonological processing is modulated by task nature. Network responses induced by C-tDCS across phonological processing tasks matched predictions. A-tDCS effects were attributed to optimisation of network efficiency.

## Introduction

Transcranial direct current stimulation (tDCS) is a brain stimulation tool that has been widely used in research with humans (Nitsche et al. 2008). However, it still yields inconsistent results across studies, especially in those studying cognition (Jacobson et al. 2012). Rodrigues de Almeida et al. (2019) pointed out that this could occur because aspects that are crucial for tDCS to have an effect in cognition have been largely overlooked. We previously discussed, under what we call the “multi-node framework”, the relevance of two aspects in particular, namely task load and the network structure underlying cognitive functions.

Regarding task load (see Rodrigues de Almeida et al. 2019 for a more comprehensive account), the effects of tDCS positively relate to the relevance of the task for the target of stimulation (which we call “task load”). Functional targeting (Bikson and Rahman 2013) needs to be satisfactory for tDCS to be effective. This means that only neurons sufficiently engaged during the task will respond to tDCS (Bikson and Rahman 2013; Fritsch et al. 2010; Meinzer et al. 2012, 2013; Pope et al. 2015) and that anatomical targeting alone is insufficient to determine the outcomes (Bikson and Rahman 2013). An increasing body of experimental studies (e.g. Meinzer et al.’s 2012, 2013) supports the concept of functional targeting. In Meinzer’s work, neuroimaging with fMRI showed that only the brain region underlying the most challenging aspect of the task responded to tDCS, despite the fact that the stimulation electrode covered neighbouring brain regions that also took part in the task.

It is also important to consider that a target in cognition normally belongs to a large brain network of nodes, which is an aspect that may further modulate tDCS effects. Network nodes may have different weightings for a task (e.g. Hartwigsen et al. 2012, 2016; Rodrigues de Almeida et al. 2019), and target responses to tDCS are expected to be more noticeable when these targets are nodes of higher weighting (Nozari et al. 2014; Pope et al. 2015). However, network responses may also arise when the target is a node of lower weighting. This is expected when a low weighting node is downregulated, as in this case there is enough room for compensation by other nodes (Rodrigues de Almeida et al. 2019). Evidence for network compensation in cognitive functions has been observed in the brain stimulation literature through behavioural and neuroimaging findings. For example, compensation can be inferred from behavioural findings where a satisfactory level of performance has been achieved despite the brain stimulation downregulating neuronal activity (e.g. Hartwigsen et al. 2013; Nozari et al. 2014; Rodrigues de Almeida et al. 2019). Compensation can also be inferred from neuroimaging findings, when increased activation of nontarget regions is reported (e.g. Hartwigsen et al. 2013).

Participation of the motor system in speech perception (Liberman and Mattingly 1985) is a claim largely supported by neuroimaging and brain stimulation studies, which show the left inferior frontal gyrus (LIFG) or the motor cortex to be involved in tasks of phonological processing (e.g. Fiez et al. 1995; Liebenthal et al. 2013; Lee et al. 2012; Meister et al. 2007; Rogers et al. 2014; Saur et al. 2008; Smalle et al. 2015; Watkins and Paus 2004; Wheat et al. 2010; Wilson et al. 2004; Woodhead et al. 2014). It is generally well accepted that phonological processing involves the analysis of both auditory and articulatory properties of speech, supported by the endpoints of the dorsal pathway, the left superior temporal gyrus (LSTG [Liebenthal et al. 2013; Saur et al. 2008]), and the LIFG (Amunts et al. 1999; Burton 2001; Cornelissen et al. 2009; Fiez et al. 1995; Indefrey 2011; Lee et al. 2012; Liakakis et al. 2011; Okada and Hickok 2006a, b; Watkins and Paus 2004; Wheat et al. 2010; Woodhead et al. 2014). With regard to task load (a point related to the current study), neuroimaging findings suggest that LIFG recruitment increases in phonological processing tasks from speech perception to speech production (Chang et al. 2010; Leonard and Chang 2014; Amunts et al. 1999; Eickhoff et al. 2009; Indefrey 2011; Liakakis et al. 2011).

Previously, Rodrigues de Almeida et al. (2019) conducted a series of behavioural experiments with tDCS of the pars opercularis (motor portion of the LIFG) to investigate the observation that task load (or relevance of the task for the target) modulates the effects of tDCS on the target region (i.e. pars opercularis of the LIFG) during a variety of phonological processing tasks (categorical perception, lexical decision and word naming) with different motor requirements. Speech perception was considered to pose the least weighting for the LIFG (Lee et al. 2012; Liebenthal et al. 2013), while speech production was considered to pose the most weighting for the LIFG (Amunts et al. 1999; Eickhoff et al. 2009; Indefrey 2011; Liakakis et al. 2011). Lexical decision was considered to occupy an intermediate weighting position for the LIFG, but rather serving as a speech perception task (i.e. posing less weighting to the target). This is because it lacks a core feature of speech production, namely overt articulation (Leuthardt et al. 2012), despite sharing features such as word recognition and orthographic to phonemic conversion with the word naming task (Burton 2001; Cornelissen et al. 2009; Deng et al. 2012; Wheat et al. 2010; Woodhead et al. 2014). Real words and nonwords were also investigated, with real words considered to pose less weighting for the LIFG (Binder et al. 2003; Fiebach et al. 2002; Forster and Chambers 1973; Levy et al. 2009; Marshall and Newcombe 1973; Patterson and Shewell 1987) than nonwords (Heim et al. 2005; Nosarti et al. 2010; Xiao et al. 2005). Predictions were that inhibition via cathodal tDCS and facilitation via anodal tDCS would have a positive relation with task load. Cathodal tDCS was also expected to induce facilitation via triggering network compensation, which should be negatively related to task load. Analyses conducted under the “multi-node framework” revealed that task load for the target of stimulation modulated the effects of tDCS effects on behavioural performance. However, the neural mechanisms proposed by the multi-node framework could not be investigated in our previous behavioural study.

The present study aimed to investigate with simultaneous tDCS and fMRI whether the task load modulation of tDCS effects observed behaviourally in Rodrigues de Almeida et al. (2019) for motor participation in phonological processing translates into a predictable pattern of functional network connectivity. The same phonological processing tasks (as in Rodrigues de Almeida et al. 2019), adapted for fMRI (see Methods), were used, as well as a similar design, with the same target of stimulation (i.e. the LIFG). The target network subserving phonological processing consisted of a restricted set of four regions of interest (ROIs) whose choice was motivated by the literature. The two endpoints of the dorsal pathway for phonological processing (i.e. the LIFG [pars opercularis] and the LSTG [Liebenthal et al. 2013; Saur et al. 2008]) were an obvious first choice. Their right homologues (i.e. the right inferior frontal gyrus [RIFG] and right superior temporal gyrus [RSTG]) were also included. These two ROIs were included because it has been shown that right homologues of left language areas are often recruited when dysfunction in the left hemisphere needs to be compensated. This can be seen in aphasia (Dominguez et al. 2014; Vitali et al. 2007), dyslexia (Pagnotta et al. 2015; Sun et al. 2010; Waldie et al. 2013), age-associated cognitive decline (Meinzer et al. 2009, 2013), task difficulty (Gur et al. 2000) and in response to perturbation via transient brain stimulation (Hartwigsen et al. 2013). Connectivity analyses in the present study were conducted with partial correlations, a standard technique for analysing functional connectivity (Marrelec et al. 2006; Ryali et al. 2012; Sandberg 2017).

**Fig. 1:**
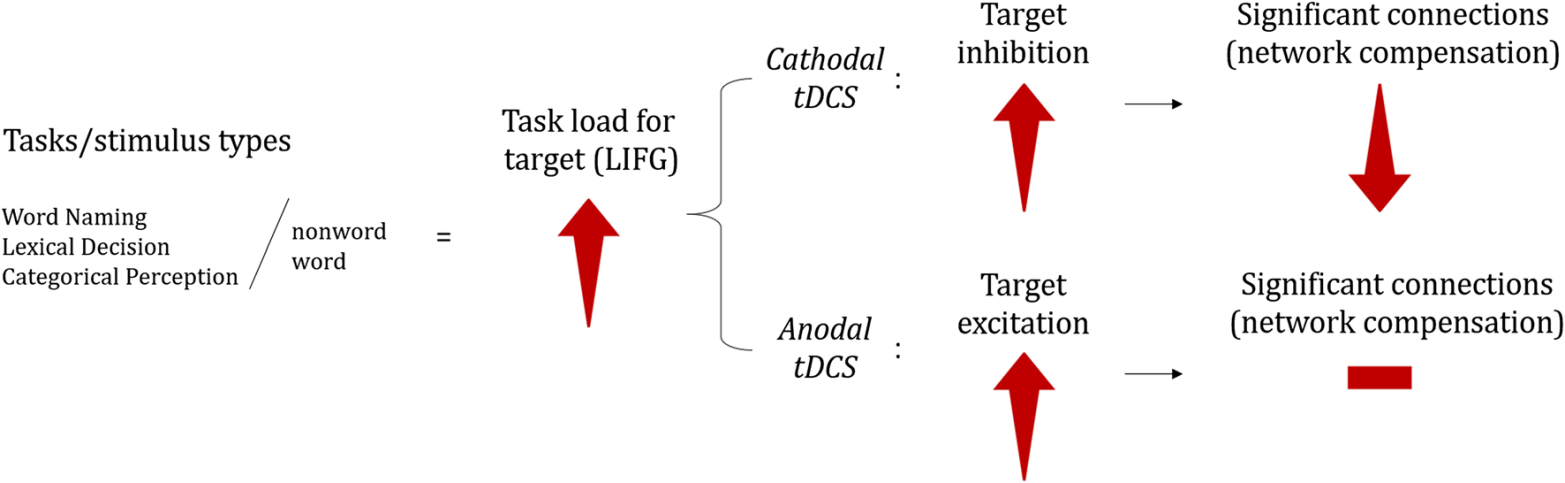
Predictions on network responses to tDCS of the LIFG during phonological processing by task and stimulus type

Our predictions are shown in Fig. 1. The modulatory role of task load on tDCS effects was predicted by comparing the relative number of significant connections within the network that anodal and cathodal tDCS of the LIFG should induce relative to sham for each phonological processing task or stimulus type condition (word vs. nonword). We predicted the network activity expected to effectively occur under each condition, and not activity ideally required for performance not to be affected by tDCS perturbation. (We elaborate on this in the next paragraph.) We assessed the number of significant network connections regardless of the potential variability in connection node identities across the different conditions, as we were interested in evaluating the target *network* response globally, rather than the response of local connections across conditions. For example, we predicted that inhibition of the LIFG (via cathodal tDCS) during categorical perception (a condition of low task load) should induce more compensation than during word naming (a condition of high task load), which should translate into a larger count of significant connections for categorical perception than for word naming. Anodal tDCS facilitation and cathodal tDCS inhibition of the target were expected to relate positively to task load. Thus, inhibition via cathodal tDCS was expected to increase with task load, while its ability to trigger compensation was expected to decrease (i.e. inducing significantly less network connections). Excitation via anodal tDCS was not expected to induce compensation.

As introduced in our previous paper (Rodrigues de Almeida et al. 2019), nodes within a network are expected to adjust and overperform if needed, as an attempt to carry out the task satisfactorily (Hartwigsen et al. 2013; Pirulli et al. 2014) despite, for example, downregulation via cathodal tDCS. Our model makes directional predictions about possible adjustments of network nodes during task load modulation of tDCS effects. That is, we predict the effective responses that the target network is able to produce after a (tDCS) perturbation, and not the ideal responses that would be required to avoid a decrease in performance caused by the perturbation. The ideal model and the effective model represent two different outcome perspectives of the same event: a perturbation to the network. For example, downregulation of a target node that is highly relevant to the task would, according to the ideal model, require more compensation for recovery than a target node that is less relevant to the same task. However, compensation for a relevant node will be unsuccessful if provided by less relevant nodes (task-irrelevant nodes) and successful if provided by more relevant nodes (task-relevant nodes). As a result, compensation when a less relevant node faces downregulation should be more successful and may result in enhanced task performance. The rationale for an effective model, which is different from the ideal model, was first inspired by findings from the tDCS literature where behavioural facilitation has been found in conditions of cathodal stimulation, often called paradoxical results because cathodal tDCS is expected to decrease performance (Jacobson et al. 2012). The effective model we previously proposed (Rodrigues de Almeida et al. 2019) is informed by work from Nozari et al. (2014). Briefly, successful compensation of inhibition via cathodal tDCS (inferred by no decrease in performance) was observed in conditions of low task load. In contrast, decreased performance was observed for conditions of high task load, suggesting that more compensation was required for recovery (i.e. the ideal model), but could not effectively take place (i.e. the effective model).

Our model specifically predicts that a node for which the task load is low has more room to be compensated from a downregulation (via cathodal tDCS) than a node for which the task load is high. This means that a node with “low task load” has a greater chance to be successfully compensated (task satisfactorily accomplished) by other, more relevant nodes in the network, because these task-relevant nodes have not been downregulated.

In contrast, when a node with “high task load” is downregulated, chances that such a node will be successfully compensated by other network nodes that are less relevant for the task (task-irrelevant nodes) are much smaller. This is because the contribution from task-irrelevant nodes to help compensate for the downregulation of a “high task load” node is insufficient to satisfactorily avoid the global decrease in task performance. Thus, task-irrelevant nodes do not compensate well for the role of a core node for the task at hand. This idea is demonstrated in the schematic representation (Fig. 1) of network activity in responses to cathodal tDCS, such that the greater the task load for the target node (LIFG), the larger the expected downregulation (i.e. inhibition), and the less successful is the compensation from other network nodes. For our tasks and stimulus types, task load on the LIFG was expected to increase from categorical perception to lexical decision to word naming, and from words to nonwords, corresponding to the increase in motor participation in phonological processing from speech perception to speech production. Correspondingly, downregulation of the target should increase across these tasks and stimulus types, with successful compensation by other network nodes decreasing. For example, categorical perception should have the highest chance of triggering compensation to overcome the downregulation of the LIFG (since it poses the least task load to the target). Indeed, we previously observed increased performance (i.e. decreased latency) for categorical perception during cathodal stimulation, suggesting successful compensation (Rodrigues de Almeida et al. 2019).

Anodal tDCS was not expected to trigger compensation (because it induces facilitation). Thus (as illustrated in Fig. 1), our model does not make predictions about network compensation in responses to anodal tDCS. However, because tDCS is dependent upon the level of engagement of the target with the task during stimulation (Bikson and Rahman 2013; Fritsch et al. 2010), our model predicts that the level of facilitation or target excitation is directly related to task load (Fig. 1). For our tasks and stimulus types, this means that excitation of the target was expected to increase from categorical perception to lexical decision to word naming, and from words to nonwords, corresponding to the increase in motor participation in phonological processing from speech perception to speech production. As for anodal tDCS of the LIFG during categorical perception, this was not expected to elicit facilitation, because task load was possibly too low to elicit a noticeable facilitatory response. Indeed, we previously observed that behavioural improvement (for latency) in categorical perception during anodal tDCS was less noticeable than that during cathodal tDCS, consistent with our prediction that compensation would be stronger for conditions of low task load (see Rodrigues de Almeida et al. 2019).

Neither the ROIs mean level of activation, nor the direction of relationships between nodes expressed by the sign of the correlation coefficients, nor the magnitude of the coefficients as a measure of connection strength was considered for analyses, as they were judged to be difficult to interpret or potentially misleading. This is because a similar pattern of brain activation (observed as BOLD signal change with fMRI) may arise for different reasons. For example, increased BOLD signal change may be observed in the target region as a result of cathodal tDCS-induced inhibition (Antal et al. 2012), but also in nontarget regions, as a result of increased excitation to compensate for the downregulation of the target (Hartwigsen et al. 2013). In addition, differences in the scale of possible change of each task could render these measurements incomparable across tasks (see Section “Regression: effects of tDCS and ROI on mean brain activation per task” for a discussion).

Effective connectivity analyses could potentially provide the predominant pattern of connection strength, as it estimates direct connections [i.e. causal relationships between every two nodes (e.g. a connection between the LIFG and LSTG or vice versa (Friston 2011))]. However, an effective connectivity analysis did not suit our study because we had no directional hypotheses, required to set up causal models for evaluation. Instead, our aim was to evaluate global changes in network activity without constraining the search to specific connections or node directions. We performed partial correlation analyses and assessed the expected increase in network activity due to compensation through the relative comparison of the number of significant connections which arose under each tDCS/task condition relative to sham. Our understanding is that those connections which appeared significant when compared to sham represent an increase in the network activity in response to tDCS stimulation modulated by task load. The larger the compensation a given condition was expected to trigger, the higher the number of significant connections that should arise relative to sham. Our procedure is equivalent to the construction of binary adjacency matrices, typically used in the first step of graph theory analyses for the identification of relevant connections for later steps of analyses (Bullmore and Bassett 2011; Farahani et al. 2019; Wang et al. 2019).

In binary adjacency matrices, the existence of connections is decided based on a threshold applied to the chosen measure of association (for example, correlation coefficients) between nodes, which will retain only the significant connections for a given condition and network of interest. Note that the count of significant connections adopted in this study allows an evaluation of global patterns of change in network activity across tasks despite any differences in scales of possible change of the individual tasks or any variability in the specific significant connections concerned. This type of measurement is ideal for evaluating the directional global predictions that our model makes for changes in network activity induced by tDCS modulated by task load. That is, increases in network activity may be revealed and compared across tasks without being masked by the magnitude of change in connection strength, which could vary greatly across tasks.

It may be worth mentioning at this point how tDCS administered with a concurrent task and how network compensation have been previously reported to translate into BOLD signal change. The BOLD signal change observed for tasks performed under tDCS may appear at first counterintuitive. The local effects of tDCS on the target are decreased BOLD signals for excitatory stimulation [i.e. with anodal tDCS (Antal et al. 2011; Fiori et al. 2018; Holland et al. 2011; Meinzer et al. 2012)] and increased BOLD signal for inhibitory stimulation [i.e. with cathodal tDCS (Antal et al. 2012)]. Although this pattern of effects seems to contradict the expected outcome, of facilitatory and inhibitory stimulation, it can be explained in terms of changes in neuronal efficiency (Holland et al. 2011). In their study, Holland and coworkers empasised that the concurrent presentation of a task during anodal tDCS may be critical to maximally facilitate task-induced neurons depolarisation, which produces less synaptic activity needed to reach a threshold. Consequently, the BOLD signal decreases. The opposite should apply to cathodal tDCS, with more synaptic activity needed to offset downregulation and reach a threshold, resulting in increased BOLD signal change. In other words, a decrease in BOLD signal change is linked to improved efficiency of the target to perform the task, while an increase in BOLD signal change is related to decreased efficiency of the target to perform the task. This pattern of BOLD signal changes in response to tDCS with a concurrent task parallels the effect of cognitive effort on the BOLD signal (Dunst et al. 2014; Engström et al. 2013), where anodal tDCS would reduce cognitive load (Fiori et al. 2018), and cathodal tDCS would increase cognitive effort. In line with this reasoning, participation of nontarget nodes for compensation of a downregulated target should translate into an increase in BOLD signal change, as it corresponds to extra nodal activity.

We note that the current work has been conducted following the same methodology and the same rationale concerning the multi-node framework as reported in Rodrigues de Almeida and Hansen (2019). However, that paper reported a pilot study with participants with dyslexia, where the language disorder was considered to modulate brain state and consequently tDCS effects on functional network connectivity. In contrast, the current study investigates nondyslexic adults, aiming to provide evidence for task load modulation of tDCS effects on functional network connectivity in the healthy brain, which could inform future work on language disorders such as dyslexia or aphasia.

## Methods

### Participants

Twenty healthy, right-handed [as assessed by Annett (1972) handedness inventory] young adults who were native English speakers took part in the study (mean age 20.5 years, SD 2.35, 9 females). All participants were assessed with the TIWRE (Reynolds and Kamphaus 2007) and TOWRE (Torgesen et al. 1999) reading tests, and none was excluded due to reading difficulties. Participants completed safety questionnaires to unsure that they were eligible to undergo tDCS and MRI. All participants gave informed consent before taking part, and the study was approved by the Central Ethics Committee of the University of Birmingham.

### Materials

#### Tasks and stimuli

The tasks used in this study are the same as those reported in Rodrigues de Almeida et al. (2019), but adapted for fMRI. Adaptations consisted of a smaller number of stimuli, variable inter-trial intervals (ITI) and specialised equipment to present stimuli and to record participants’ responses. Behavioural data were not suitable for analyses, due to the smaller number of stimuli used than in our previous study, and technical issues with the collection of oral responses in the word naming task (see explanation below). Therefore, behavioural responses in this study were only logged to confirm task compliance. A full account of task load modulation of tDCS effects on behavioural performance can be seen in our previous study (Rodrigues de Almeida et al. 2019). In this paper, there is also a fuller description of the stimuli. Table 1 shows summary statistics of preprocessed measurements of behavioural performance by task and tDCS condition from that paper for the reader’s reference.

**Table 1:** Delta (run 2–run 1) latency and accuracy by task and tDCS condition: summary statistics of preprocessed behavioural data from Rodrigues de Almeida et al. (2019)

Categorical perception	Lexical decision			Word naming		
	Task	Words	Nonwords	Task	Words	Nonwords
*RT* (ms)						
A						
− 8.54(54.06), − 147.86 to 79.21	− 3.20(46.80), − 93.32 to 83.70	− 7.82(44.76), − 91.73 to 76.95	− 0.56(54.87), − 96.12 to 82.28	5.05(22.09), − 34.43 to 44.40	3.53(23.25), − 39.55 to 44.77	6.48(23.22), − 31.75 to 44.03
C						
− 15.39(50.50), − 164.03 to 97.32	− 11.70(44.13), − 135.50 to 52.35	2.21(51.33), − 94.69 to 102.07	− 27.07(50.42), − 191.06 to 68.30	8.52(49.36), − 69.49 to 134.10	7.74(48.39), − 66.01 to 121.85	8.37(51.39), − 77.41 to 137.45
S						
6.01(50.59), − 105.64 to 86.72	− 2.98(78.26), − 254.03 to 116.60	10.03(60.42), − 117.27 to 104.75	− 15.82(101.87), − 378.50 to 137.99	16.86(36.97), − 40.19 to 85.64	20.31(40.16), − 46.07 to 103	13.26(35.42), − 42.86 to 78.05
*ACC*^a^						
A						
− 0.72(0.90), − 2.57 to 0.67	− 0.01(0.04), − 0.11 to 0.10					
C						
− 0.33(0.82), − 2.11 to 1.64	− 0.02(0.04), − 0.08 to 0.07					
S						
0.10(0.41), − 0.59 to 1.13	0.00(0.05), − 0.07 to 0.08					

Cells contain: mean(standard deviation), range

*RT* reaction times, *ACC* accuracy, *A* anodal tDCS, *C* cathodal tDCS, *S* sham

^a^Unit for ACC in categorical perception is slope for uncertainty (refer to Rodrigues de Almeida et al. 2019 for details)

The categorical perception task involved the judgment of ten 300 ms speech sound tokens from a continuum of synthesised speech between /ba/ and /da/, which should be categorised as either one of the endpoints. We used stimuli as reported in the study by Raizada and Poldrack (2007), which are detailedly described in their paper. The continuum was generated with a SenSyn Klatt synthesiser through the manipulation of the second and third formants of the endpoints. The sound tokens were each repeatedly presented in randomised order for an unequal number of times. The two more extreme tokens corresponding to the endpoints were presented 30% of the times, and the remaining tokens, which were more challenging to categorise, were presented 70% of the time. An MRI compatible headset (ConFon Electro Dynamic Headphones; MR confon GmbH, n.d.) was used to deliver the sounds. Participants made their judgment by pressing the corresponding button with the left hand using a button box.

For the lexical decision and word naming tasks, an equal number of words and nonwords were randomly presented on the screen for 500 ms each per run. Stimuli were presented between two aligned vertical bars that stayed visible throughout the run. Different, but matched, lists of stimuli were used between the two runs and were the same for both tasks. As detailed in Rodrigues de Almeida et al. (2019), six letter words and nonwords were generated for the lexical decision and word naming tasks. Words were generated with the VWR R package (Keuleers 2013) and controlled for orthographic neighbourhood density (OLD20 score; Yarkoni et al. 2008) and frequency (SUBTLEXus database of word frequency for American English; Brysbaert and New 2009). Nonwords were generated with the Wuggy pseudowords generator (Keuleers and Brysbaert 2010) and matched to the list of words by OLD20 score.

In the lexical decision task, participants judged whether the stimulus was a real word or a nonword by pressing the corresponding button with the left hand. In the word naming task, all stimuli presented should be read aloud as promptly as possible. Voice responses were recorded with an MRI compatible microphone (Optoacoustics’ FOMRI III + Noise Cancelling microphone; Optoacoustics Ltd., n.d.). However, it was not possible to filter the voice responses from the scanner noise, and therefore, the voice onset times could not be used in the analyses.

We used a rhyme judgment task for the initial period of stimulation with tDCS to ensure enough time for tDCS to start to have an effect (Nozari et al. 2014) before the experimental tasks were presented. We chose a rhyme judgment as the “warming-up” task because tDCS effects are sensitive to the engagement of the target with the task at hand (Bikson and Rahman 2013; Fritsch et al. 2010). Therefore, a task involving phonological processing and hence recruitment of the LIFG (Burton et al. 2003; Zhu et al. 2016) was needed. Its task load for the LIFG was believed to be rather low, as it would fall more towards the speech perception end of the speech perception to speech production range, due to the lack of overt articulation, a core feature of speech production (Leuthardt et al. 2012). However, the possibility of a carry-over effect from the rhyme judgment to the experimental tasks, potentially different across tasks of different loads for the LIFG, could not be ruled out. To minimise the potential differential effect of an interaction between the rhyme judgment and the experimental tasks, we counterbalanced task order presentation across participants with a Williams design [Williams 1949 (detailed in Rodrigues de Almeida et al. 2019)]. No imaging data were acquired during this task. The rhyme judgment task used in the current study was identical to that used in Rodrigues de Almeida et al. (2019), consisting of the same subset of McNorgan and Booth (2015)’s list of pairs of words, where only half rhymed. Eighty pairs of words were randomly presented on the screen for 900 ms between aligned vertical bars for each pair. Participants should judge each word pair as a pair that rhymed or a pair that did not rhyme by pressing the corresponding button with the left hand. A single run of this task was presented between the two blocks of experimental tasks with an inter-trial interval of 2 s.

All experimental tasks (categorical perception, lexical decision and word naming) had 60 stimuli per run and two runs per session [each session corresponded to one tDCS condition (i.e. anodal, cathodal or sham)]. Stimuli were presented with a variable inter-trial interval that followed a Poisson distribution whose mean was of 9.5 s. Visual stimuli were delivered with the Presentation software (version 18.3, Neurobehavioral Systems) via projector during the fMRI sessions.

#### tDCS

Direct current with 2 mA of intensity was delivered with an MRI compatible neuroConn tDCS device (neuroConn GmbH, n.d.) through square rubber electrodes measuring 25 cm^2^ each. With assistance of an EEG cap, the active electrode was placed on the LIFG, F5 according to the 10–20 international EEG system (Jasper 1958), and the reference electrode was placed over the contralateral supraorbital region. Real tDCS conditions had a duration of stimulation of 20 min. The sham condition lasted 30 s including a ramp-up and a ramp-down period of current delivery. This is within the typical range of duration that is not enough to modulate brain function and therefore ensures a satisfactory placebo for sham stimulation (Nitsche et al. 2008). Real stimulation started with the direct current increasing from zero to 2 mA with a ramp of 10 s and finished by decreasing the current from 2 mA to zero with a ramp down of 10 s. Our participants often queried the researchers conducting the session as to know which of the sessions presented a real stimulation condition and which a sham stimulation condition. (This information was available to them after the third session.) This suggests that all conditions were sufficiently similar in terms of skin sensations, so that participants were blind with respect to which condition they were in. This further indicates that a similar sensation at the beginning of stimulation is the crucial factor to mask conditions, and more importantly, participants notice the ramp down of 20 min of real stimulation due to habituation. However, this conclusion is based on reports from a subset of participants. We did not collect data about participants’ (possible) discomfort to stimulation for each session as to evaluate this matter more precisely.

Ten20 conductive paste was applied to the electrodes to reduce scalp electrical resistance. This paste was used in lieu of the typical saline solution to avoid drying out of the electrodes during the experiments, since participants would be wearing them for a long time before the brain stimulation started.

### Procedure

The experiments were run with a within-subject design and a single blind protocol, where participants were unaware of the tDCS condition of each session. TDCS conditions of anodal, cathodal and sham were presented in different sessions and counterbalanced across participants. Time between sessions was variable, depending on participant and scanner availability (mean = 53.78 days, range = 7–288). The average duration between sessions was therefore greater than the standard one week interval typically reported in the literature to avoid spillover effects of tDCS from one session to another (Woods et al. 2016).

Participants practiced the experimental tasks and the rhyme judgment task prior to the experimental sessions. Written instructions were presented on screen before each task and orally reinforced by the researcher during the session. Participants were reminded to respond to each task as quickly as possible.

#### Design of the experimental sessions

**Fig. 2:**
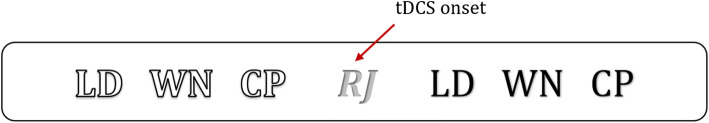
Schematic representation of an experimental session. Experimental tasks are represented in black with unfilled text font for the baseline runs and with filled text font for the online (during tDCS) runs. The “warming-up” task is represented in grey. *CP*, categorical perception; *LD*, lexical decision; *WN*, word naming and *RJ*, rhyme judgment

Task order presentation was counterbalanced across participants with a Williams design (Williams 1949) (detailed in Rodrigues de Almeida et al. 2019) to minimise a potential carry-over effect, but was kept the same across the two blocks of a session and across all the sessions of each participant. Categorical perception, lexical decision and word naming were presented in two blocks of scans per session, one block of scans for baseline and the other one during brain stimulation (online run or repeat). The rhyme judgment task was presented between the two blocks of scans, in the beginning of the tDCS (Fig. 2). The effects of the current were assumed to be the same for all the experimental tasks of the online run, since the effects of the current have already been shown in the literature to persist for minutes after the end of the stimulation (Mangia et al. 2014). FMRI was acquired during the experimental tasks, with one scan per task. The rhyme judgment was run without scanning. A structural anatomical scan of each participant was acquired after the experimental runs of any one of the three tDCS sessions.

#### MRI acquisition parameters

A 32-channel head-coil 3T Philips Achieva scanner was used to collect MRI data at the Birmingham University Imaging Centre (BUIC). Two hundred and forty T2*-weighted gradient-echo EPI volumes were acquired per scan (or experimental run), with a repetition time (TR) of 2.5 s, echo time (TE) of 34 ms, flip angle (FA) of 77o, slice thickness of 3 mm, voxel size of 3 mm^3^, field of view (FOV) of 240 × 130 × 240 mm and acquisition matrix of 80 × 80. Each EPI volume had 43 axial oblique slices, which was enough to cover the whole cortex. Slices were acquired in sequential descending order. There is some recommendation in the literature for the use of sparse sampling for speech production and speech perception tasks in order to minimise motion artefacts caused by articulation and the interference of background scanner noise with the reception of speech (Raizada and Poldrack 2007; Ulm et al. 2015). However, this would bring the caveat of sequence variability between tasks, as well as diminished statistical power, due to the reduction in number of stimuli to fit the task within the same duration. To avoid these issues, the typical nonsparse sampling sequence was equally used for all the tasks. A pilot study was run to ensure the feasibility of the experimental tasks with the typical sequence. In particular, to ensure that the auditory stimuli were sufficiently audible over the scanner noise for meaningful completion of the categorical perception task, we used a special set of MRI compatible headphones [ConFon Electro Dynamic Headphones (MR confon GmbH, n.d.)]. This type of headphones uses the magnetic field of the scanner to adjust the filtering of the noise, resulting in superior performance compared to typical MRI compatible headsets without this technology. We performed testing sessions using these headsets with two native English speakers, who confirmed that they could clearly hear the stimuli for meaningful task completion. The structural anatomical scan was an isotropic T1-weighted gradient-echo image with the following parameters: 175 sagittal slices, TR = 8.4 ms, TE = 3.8 ms, flip angle of 7° and voxel size of 1 mm^3^.

### Analyses

#### Preprocessing

The FMRIB Software Library (FSL; Jenkinson et al. 2012; Smith et al. 2004; Woolrich et al. 2009) was used for preprocessing of functional and structural images and to analyse the fMRI data. Nonbrain tissue was removed from structural anatomical images (T1) with the FSL BET (v.2.1) tool (Smith 2002). Functional images received regular-down slice timing correction. Images were spatially smoothed with a Gaussian kernel of 4.5 mm (1.5 times one dimension of the isotropic 3 mm3 voxel). Motion correction of the functional data was performed by using the MCFLIRT tool (Jenkinson et al. 2002) and the ICA-AROMA (ICA-based Automatic Removal Of Motion Artifacts) tool (Pruim et al. 2015a, b). The MCFLIRT applied rigid body transformation with the middle image as reference. The ICA-AROMA was used to identify and remove motion-related ICA components from the data. Temporal filtering was applied after ICA-AROMA motion correction. The high-pass Gaussian-weighted filter cut-off was of 50 s.

Multi-stage registration was performed to align functional to structural images. A 6-DOF affine registration was used to register functional images to individual anatomical space with the FSL FLIRT tool (v.6.0) (Jenkinson and Smith 2001; Jenkinson et al. 2002). A nonlinear registration (warp resolution 10 mm) of each functional image into standard MNI space was then performed with the FSL FNIRT tool (Andersson et al. 2007a, b).

#### Data analyses

##### Step of whole brain analyses

Whole brain analyses were conducted as a first step of analyses before mean activation of regions of interest (i.e. regions of the target network for phonological processing [LIFG, LSTG, RIFG and RSTG], could be calculated). Brain activation induced by the factors of task, tDCS and population were calculated at this stage (data not reported).

Data were analysed with the FSL FEAT v.6.0 tool (Woolrich et al. 2001, 2004). A general linear model (GLM) with local autocorrelation correction (using FILM prewhitening; Woolrich et al. 2001) was used to analyse all conditions at the individual level. Each of the functional scans in a session corresponded to one task (either categorical perception, lexical decision or word naming), one tDCS condition (either anodal, cathodal or sham for a particular session, or day of data collection, which itself contained six scans) and one repeat or run (either baseline or online). In the first level of analysis, only task was therefore modelled as factor of interest for each scan.

For the lexical decision and the word naming tasks, different stimulus types were entered into the design matrix as separate covariates (i.e. words and nonwords) were modelled separately. The onset of responses was included in the design matrix as nuisance covariates whenever available (not available for word naming). Stimuli presentation and responses had their onset and duration modelled. Button responses were given a notional duration of 100 ms. Time courses associated with each event where onset and duration were modelled were convolved with a double-gamma HRF (hemodynamic response function). Temporal filtering was applied and temporal derivatives were added to the model as separate nuisance covariates in order to improve the model fit. Motion parameters generated by MCFLIRT were also included as nuisance covariates to regress out unwanted influence of motion on performance (Johnstone et al. 2006). T-contrasts were generated for the mean of all stimulus types versus rest for all the tasks. In addition, for the lexical decision and word naming tasks, t-contrasts were generated for the mean of each stimulus type, i.e. words and nonwords, versus rest.

Second-level analyses were carried out with fixed effect models with the contrast images from the first-level analyses as input [i.e. contrast images for the mean across all the stimuli of each task (and mean across stimulus types for lexical decision and word naming) per run (baseline and online) and tDCS condition (anodal, cathodal and sham)]. Each task (and stimulus type for lexical decision and word naming)/tDCS combination was set up separately as a covariate of interest in the design matrix. The difference between the online and the baseline runs was set up in the design matrix within the covariates for task/tDCS combinations. T-contrasts were set up to compare the differences between real tDCS (anodal or cathodal) and sham for each task (and stimulus type for lexical decision and word naming).

Group analyses were carried out with random effect models using FLAME stage 1 (Beckmann et al. 2003; Woolrich et al. 2004; Woolrich 2008). Gaussian random field theory thresholding was applied to the statistical maps, with a value of *Z* > 2.3 at the voxel level and *p* < 0.05 at the cluster level, corrected for multiple comparisons. *Z* value activation maps were produced for each contrast. The second-level output images of each participant entered the random effects models as input. Mean *t*-contrasts (one-sample *t* tests) were set up to analyse the group mean brain activation for each task (and stimulus type for lexical decision and word naming)/tDCS combination from the second levels.

##### Network analyses

Network analyses were conducted to investigate whether task load modulation of the effects of tDCS on the target (LIFG) could be inferred from functional network connectivity patterns in the target network for phonological processing (consisted of LIFG, LSTG, RIFG and RSTG).

##### ROI definitions and ROI-based data measurements

The network of interest consisted of the two typical nodes involved in phonological processing, LIFG (pars opercularis) and LSTG (Burton 2001; Liebenthal et al. 2013; Saur et al. 2008), and their right homologues, RIFG and RSTG. It should be noted that the target of tDCS in this study, and therefore the selected ROI, was specifically the pars opercularis of the LIFG, the subregion reported in the literature to have a core role in phonological processing (Lee et al. 2012; Saur et al. 2008; Watkins and Paus 2004). Although neighbouring language areas were most likely affected by the tDCS current, under a functional targeting perspective (Bikson and Rahman 2013; Meinzer et al. 2012, 2013), only the pars opercularis of the LIFG should be sufficiently engaged with the most challenging aspect of the tasks (i.e. phonological processing), as to react to stimulation. Four ROIs corresponding to the four nodes of the network of interest were then created with FSL command line tools as a 6-mm-radius sphere centred at coordinates defined in MNI space.

Coordinates for the LIFG and LSTG were obtained from meta-analyses of functional brain activation associated with the categorical perception, lexical decision and word naming tasks. These were carried out using the Neurosynth software and database (Neurosynth 2018; Yarkoni et al. 2011a, b). The search for each task used, respectively, the keywords “speech perception”, “lexical decision” and “speech production” and yielded three forward inference statistical maps Benjamini–Hochberg-corrected for multiple comparisons with a threshold of *P* < 0.01 (see the Neurosynth website and Yarkoni et al. 2011a, b for further information). By using FSL command line tools, the intersection between the three statistical images was obtained. The resulting image was submitted to a cluster analysis with a *Z* threshold of 2.3. Clusters corresponding to the LIFG and the LSTG in the Harvard–Oxford cortical atlas available in FSL (Desikan et al. 2006; Frazier et al. 2005; Goldstein et al. 2007; Makris et al. 2006) were identified through their centre of gravity (COG), that is an average of the coordinates within the cluster weighted by intensity. These were then chosen as the coordinates for LIFG and LSTG. Coordinates for the right homologues RIFG and RSTG were the same as those for the left ROIs, but with the sign for the x-coordinate reversed. The MNI coordinates for the four ROIs were *x* = − 50, *y* = 14 and *z* = 24 (LIFG), *x* = − 58, *y* = − 28 and *z* = 4 (LSTG), *x* = 50, *y* = 14 and *z* = 24 (RIFG) and *x* = 58, *y* = − 28 and *z* = 4 (RSTG).

For each participant, mean percentage signal changes were obtained for each condition of interest per ROI with the FSL Featquery tool, based on whole brain analysis contrasts. Conditions of interest were the effect of task (and stimulus type for lexical decision and word naming) in combination with tDCS (second-level contrasts) on brain activation. Contrasts involving the factor tDCS were defined with run 1 (baseline) subtracted from run 2 (online stimulation) and sham subtracted from real tDCS conditions (henceforth “anodal tDCS” or “cathodal tDCS”).

##### Partial correlation: ROI-based connectivity analyses per task and tDCS combination

Correlational analyses can provide indirect measurement of functional connectivity and have been extensively used for individual-level analyses (e.g. Marrelec et al. 2006; Ryali et al. 2012; Sandberg 2017), especially when precise prior information (e.g. temporal) for the connections between pairs of nodes (usually required to perform effective connectivity analyses) is not available. Partial correlation is therefore deemed to be a reasonable option (Marrelec et al. 2006). Furthermore, as connectivity analyses in this study were based on a previously defined network, partial correlation was considered more adequate than seed-based analyses, which are rather exploratory. For these reasons, partial correlation was the analysis of choice to investigate functional connectivity in this study. We predicted that the amount of significant correlations between nodes induced by each task/tDCS condition would reveal the task load modulation of tDCS effects.

Partial correlation analyses using Pearson correlation coefficients (r), and their level of significance, were calculated for data sets of ROI mean brain activations with the PPCOR R package (Kim 2015). These analyses were performed to investigate the relationships between each pair of nodes of the target network for the different conditions of task and tDCS, to show the brain activity subserving performance. Data sets for each condition were selected according to contrasts of task or stimulus type (for lexical decision and word naming) in combination with tDCS.

##### Regression: effects of tDCS and ROI on mean brain activation per task

As referred to in Introduction, information provided by the mean level of activation for an individual ROI could be difficult to interpret because similar levels of change in BOLD signal strength may have very different underlying causes. For this reason, measurements of mean level of activation per ROI were not considered for our predictions on task load modulation of tDCS effects. The regression analyses described in this section were conducted for completeness.

ROI mean activation measurements were fed into a mixed effect linear regression analysis performed in R version 3.4.2 (R Core Team 2017) with the *nlme* package (Pinheiro et al. 2017) per task to investigate the main effects of the factors of tDCS and ROI on BOLD signal change, with participants included in the models as random effects. Additional analyses were performed for the lexical decision and word naming tasks, with the inclusion of the factor stimulus type (words and nonwords) in the model. Effect sizes for interactions and main effects were calculated with partial eta squares from *F* values and their degrees of freedom following Lakens (2013) with this formula: *η*_p_^2^ = (*F* * df_effect_)/(*F* * df_effect_ + df_error_).

Post hoc analyses per task were conducted with contrasts to investigate whether the brain activation induced by anodal and cathodal tDCS was significantly different from zero per ROI. Post hoc analyses for models which included stimulus type as a factor were conducted to investigate whether the effect of tDCS on brain activation per ROI was different from zero for each stimulus type. All post hoc comparisons were corrected for multiple comparisons using the Benjamini–Hochberg method (Benjamini and Hochberg 1995).

It should be noted that analyses by task were preferred over a single analysis with an ordinal variable where tasks and their relative loads could be included, which was technically feasible for regression analyses (but not for connectivity analysis with partial correlations, to the best of our knowledge). This methodological decision was made for consistency with our previously published behavioural paper (Rodrigues de Almeida et al. 2019), in order to avoid a potential confound. As discussed in that paper, differences in magnitude of measurements of performance may not reflect a difference in response to the experimental manipulation (e.g. a given tDCS condition), but rather a difference in the individual rate of change of each task. For example, the maximum possible change in latency for the categorical perception task could be, in the word naming or in the lexical decision scale, always placed at the lower range of possible change and therefore always appear to be smaller than for word naming or lexical decision. Thus, an improvement in categorical perception would always be considered unimportant when compared to an improvement in word naming. However, our model of connectivity stated predictions based on categorical patterns of change. According to our model, a node with a small task load was expected to show more facilitation under cathodal tDCS, than under anodal tDCS, and this was indeed the case (in Rodrigues de Almeida et al. 2019) for categorical perception, but not for word naming or lexical decision, which have higher load for the target (LIFG). With the different scales of possible raw change in latency by task as a potential confound, it would not be feasible to evaluate the model on connectivity that we set up to predict tDCS effects on behaviour, as connectivity patterns of change would be masked by these uninformative raw magnitudes of change.

We applied the same rationale discussed in our previous paper (Rodrigues de Almeida et al. 2019) to our (indirect) measurements of brain activity in the current paper. Statistical comparisons of mean levels of activation or individual connection strengths across tasks were not carried out, as a difference in the scale of change could potentially intervene as a confounder and render the results difficult to interpret. Indeed, the possibility of a confounder of difference in the scale of change due to task nature seems well appreciated in the neuroimaging literature when conducting analyses with a parametric factor, which would be the equivalent of an ordinal variable in a regression analysis, is normally only performed within the same task type. The levels of the single task type are typically different levels of difficulty or intensity of the parameter of interest (Soares et al. 2016), for example, the working memory load on n-back tasks (Jansma et al. 2004; Schmidt et al. 2009) and visual attention and encoding on visual tasks (Müller et al. 2003; Rombouts et al. 1999; Tomasi et al. 2004).

## Results

Functional network connectivity patterns resulting from tDCS of the LIFG were investigated with partial correlation analyses per task or stimulus type (for lexical decision and word naming) and tDCS condition. Effects of tDCS, ROI and stimulus type (additionally for lexical decision and word naming) on brain activation were analysed with mixed effect models per task for completeness. Both types of analyses are presented in this section by task.

### Categorical perception

#### Effects of tDCS and ROI on mean brain activation

A 2 × 4 (tDCS x ROI) linear mixed effect model was fitted to the mean parameter estimates of ROI activation data of both anodal and cathodal tDCS conditions. Neither the interaction between tDCS and ROI (*F*(3,133) = 0.38, *p* = 0.77, *η*_p_^2^ = 0.01) nor the main effects of tDCS (*F*(1,133) = 0.61, *p* = 0.44, *η*_p_^2^ = 0.005) and ROI (*F*(3,133) = 1.04, *p* = 0.38, *η*_p_^2^ = 0.02) were significant.

**Fig. 3:**
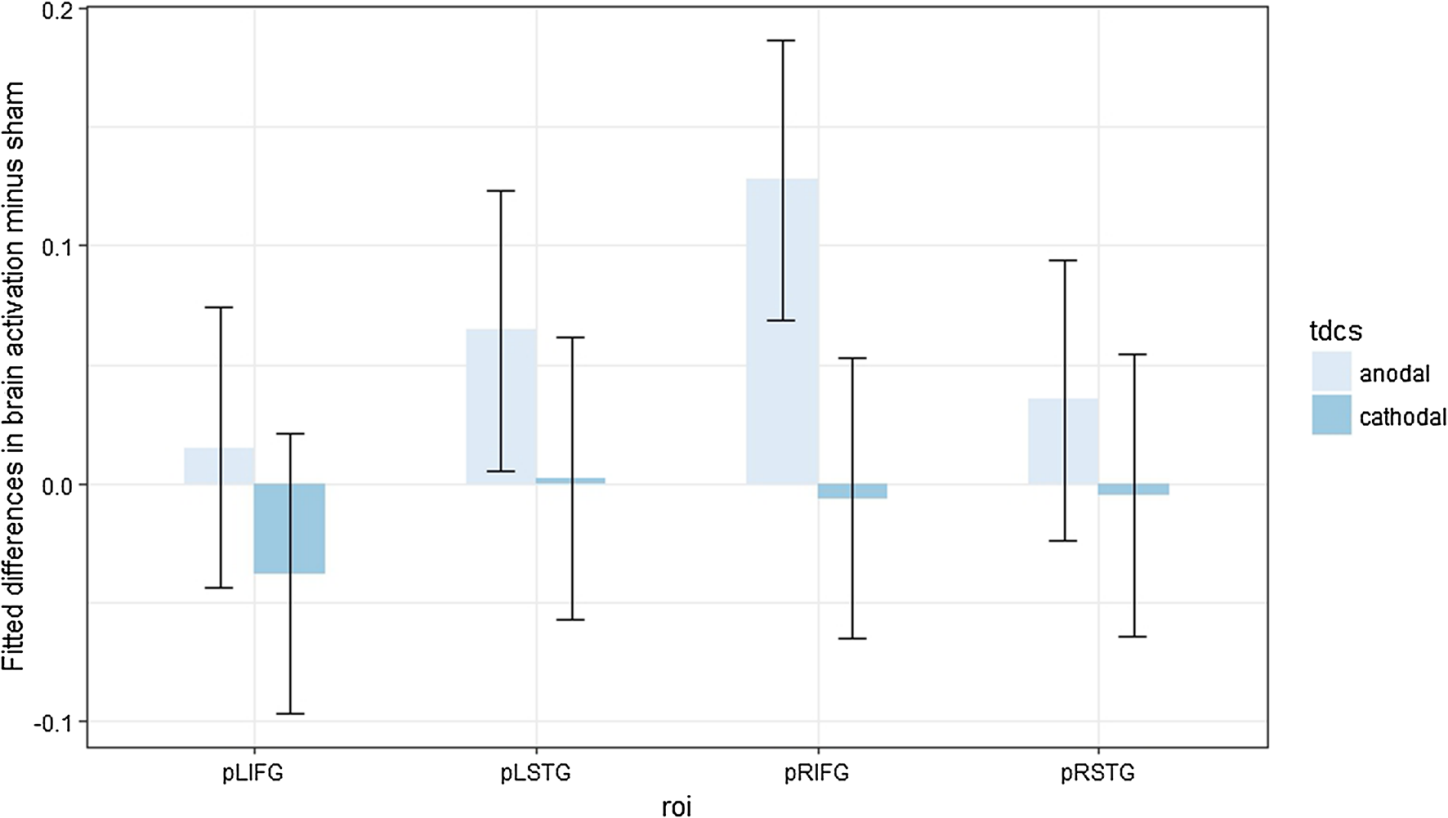
Fitted mean brain activation per ROI and tDCS for categorical perception. The *x*-axis displays the ROIs. The *y*-axis displays the fitted mean brain activation. Error bars represent the contrast estimate ± the pooled standard error

Post hoc contrast analyses (Benjamini–Hochberg-corrected for multiple comparisons) were performed, but none of them appeared significantly different from zero (Fig. 3).

#### Connectivity analysis per tDCS condition

Partial correlation analyses were performed between the fitted mean brain activations of the target network ROIs by tDCS condition. Cathodal tDCS induced a larger number of significant correlations than anodal tDCS. These occurred for the network connections LIFG/RIFG, LSTG/RSTG and RIFG/RSTG under cathodal tDCS and LIFG/RIFG under anodal tDCS (Tables 2, 3).

**Table 2:** Partial correlation analyses for fitted mean brain activations under anodal tDCS in categorical perception

	LIFG	LSTG	RIFG	RSTG
LIFG	1.00	0.35	***0.57***	0.03
LSTG	0.35	1.00	− 0.11	0.28
RIFG	0.57	− 0.11	1.00	0.36
RSTG	0.03	0.28	0.36	1.00

Pearson’s *r* for each pair of ROIs with significant correlations at *p* ≤ 0.05 marked in bold italic

**Table 3:** Partial correlation analyses for fitted mean brain activations under cathodal tDCS in categorical perception

	LIFG	LSTG	RIFG	RSTG
LIFG	1.00	0.30	***0.70***	− 0.22
LSTG	0.30	1.00	− 0.28	***0.72***
RIFG	0.70	− 0.28	1.00	***0.48***
RSTG	− 0.22	0.72	0.48	1.00

Pearson’s *r* for each pair of ROIs with significant correlations at *p* ≤ 0.05 marked in bold italic

### Lexical decision

#### Effects of tDCS and ROI on mean brain activation

A 2 × 4 (tDCS × ROI) linear mixed effect model was fitted to the mean parameter estimates of ROI activation data of both anodal and cathodal tDCS conditions. The interaction between tDCS and ROI (*F*(3,133) = 0.45, *p* = 0.72, *η*_p_^2^ = 0.01), as well as the main effect of tDCS (*F*(1,133) = 2.38, *p* = 0.13, *η*_p_^2^ = 0.02), was nonsignificant, but a significant main effect of ROI (*F*(3,133) = 4.28, *p* < 0.01, *η*_p_^2^ = 0.09) was observed.

**Fig. 4:**
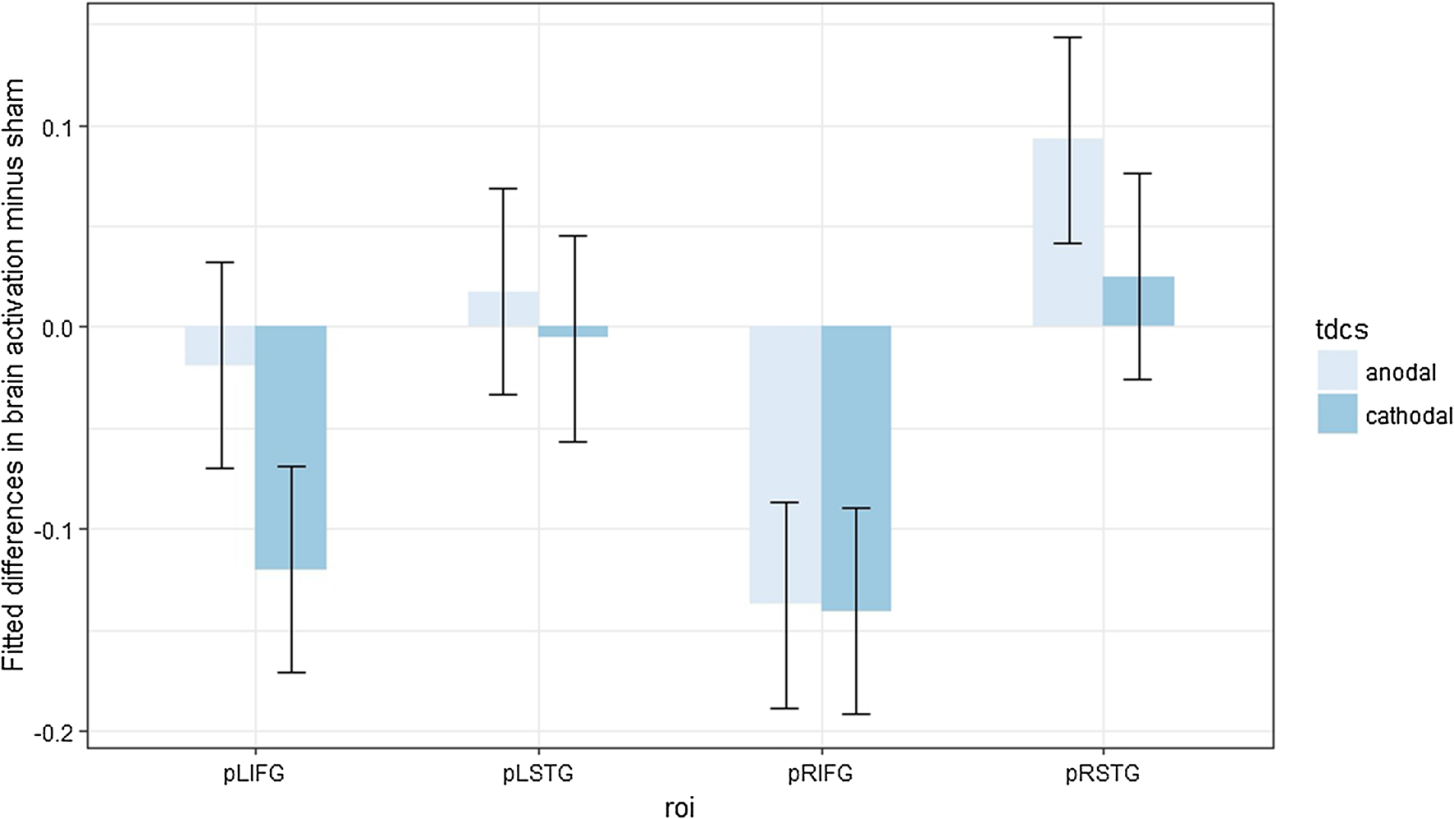
Fitted mean brain activation per ROI and tDCS for lexical decision. The *x*-axis displays the ROIs. The *y*-axis displays fitted mean brain activation. Error bars represent the contrast estimate ± the pooled standard error

Post hoc contrast analyses (Benjamini–Hochberg-corrected for multiple comparisons) were performed, but none of them appeared significantly different from zero (Fig. 4).

#### Connectivity analysis per tDCS condition

Partial correlation analyses were performed between the fitted mean brain activations of the target network ROIs by tDCS condition. Cathodal stimulation induced a larger number of significant correlations than anodal tDCS. These occurred for the network connections LIFG/LSTG, LIFG/RIFG, RIFG/LSTG, LSTG/RSTG and RIFG/RSTG under cathodal tDCS and LSTG/RSTG under anodal tDCS (Tables 4, 5).

**Table 4:** Partial correlation analyses for fitted mean brain activations under anodal tDCS in lexical decision

	LIFG	LSTG	RIFG	RSTG
LIFG	1.00	− 0.14	0.28	0.27
LSTG	− 0.14	1.00	0.28	***0.68***
RIFG	0.28	0.28	1.00	0.00
RSTG	0.27	0.68	0.00	1.00

Pearson’s *r* for each pair of ROIs with significant correlations at *p* ≤ 0.05 marked in bold italic

**Table 5:** Partial correlation analyses for fitted mean brain activations under cathodal tDCS in lexical decision

	LIFG	LSTG	RIFG	RSTG
LIFG	1.00	***0.48***	***0.83***	− 0.45
LSTG	0.48	1.00	− ***0.67***	***0.67***
RIFG	0.83	− 0.67	1.00	***0.63***
RSTG	− 0.45	0.67	0.63	1.00

Pearson’s *r* for each pair of ROIs with significant correlations at *p* ≤ 0.05 marked in bold italic

### Word naming

#### Effects of tDCS and ROI on mean brain activation

A 2 × 4 (tDCS × ROI) linear mixed effect model was fitted to the mean parameter estimates of ROI activation data of both anodal and cathodal tDCS conditions. Neither the interaction between tDCS and ROI (*F*(3,133) = 0.11, *p* = 0.95, *η*_p_^2^ = 0.003) nor the main effects of tDCS (*F*(1,133) = 0.11, *p* = 0.74, *η*_p_^2^ = 0.001) and ROI (*F*(3,133) = 0.90, *p* = 0.44, *η*_p_^2^ = 0.02) were significant.

**Fig. 5:**
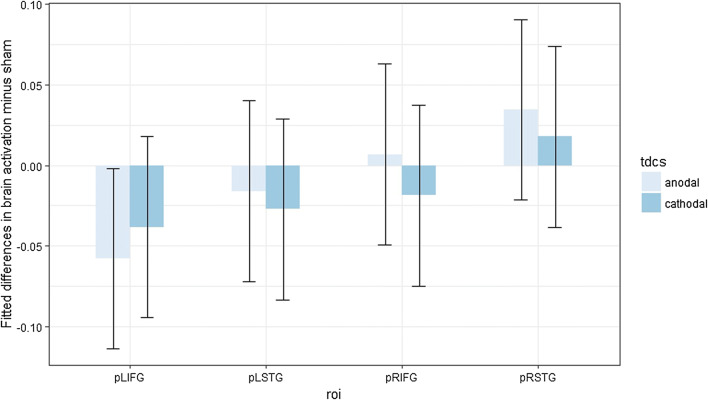
Fitted mean brain activation per ROI and task for word naming. The *x*-axis displays the ROIs. The *y*-axis displays the fitted mean brain activation. Error bars represent the contrast estimate ± the pooled standard error

Post hoc contrast analyses (Benjamini–Hochberg-corrected for multiple comparisons) were performed, but none of them appeared significantly different from zero (Fig. 5).

#### Connectivity analysis per tDCS condition

Partial correlation analyses were performed between the fitted mean brain activations of the target network ROIs by tDCS condition. Both anodal and cathodal tDCS induced significant correlations between the same pairs of ROIs: the LIFG/RIFG and the LSTG/RSTG (Tables 6, 7).

**Table 6:** Partial correlation analyses for fitted mean brain activations under anodal tDCS in word naming

	LIFG	LSTG	RIFG	RSTG
LIFG	1.00	0.18	***0.76***	− 0.07
LSTG	0.18	1.00	0.01	***0.60***
RIFG	0.76	0.01	1.00	0.22
RSTG	− 0.07	0.60	0.22	1.00

Pearson’s *r* for each pair of ROIs with significant correlations at *p* ≤ 0.05 marked in bold italic

**Table 7:** Partial correlation analyses for fitted mean brain activations under cathodal tDCS in word naming

	LIFG	LSTG	RIFG	RSTG
LIFG	1.00	0.18	***0.69***	− 0.19
LSTG	0.18	1.00	0.00	***0.52***
RIFG	0.69	0.00	1.00	0.40
RSTG	− 0.19	0.52	0.40	1.00

Pearson’s *r* for each pair of ROIs with significant correlations at *p* ≤ 0.05 marked in bold italic

### Analysis of words and nonwords in lexical decision

#### Effects of stimulus type, tDCS and ROI on mean brain activation

A 2 × 3 × 4 (stimulus type × tDCS × ROI) linear mixed effect model was fitted to the mean parameter estimates of ROI activation data of words and nonwords in the lexical decision task data of the healthy young adult sample whose target of stimulation was the LIFG. Only main effects of tDCS (*F*(1,288) = 3.88, *p *= 0.049, *η*_p_^2^ = 0.01) and ROI (*F*(3,288) = 3.91, *p *< 0.01, *η*_p_^2^ = 0.04) were significant. The main effect of stimulus type (*F*(1,288) = 0.63, *p* = 0.43, *η*_p_^2^ = 0.002) and the two-way interactions of stimulus type × tDCS (*F*(1,288) = 0.07, *p* = 0.80, *η*_p_^2^ = 0.0002), stimulus type × ROI (*F*(3,288) = 0.27, *p* = 0.85, *η*_p_^2^ = 0.003) and tDCS × ROI (*F*(3,288) = 0.78, *p* = 0.51, *η*_p_^2^ = 0.01) were nonsignificant.

**Fig. 6:**
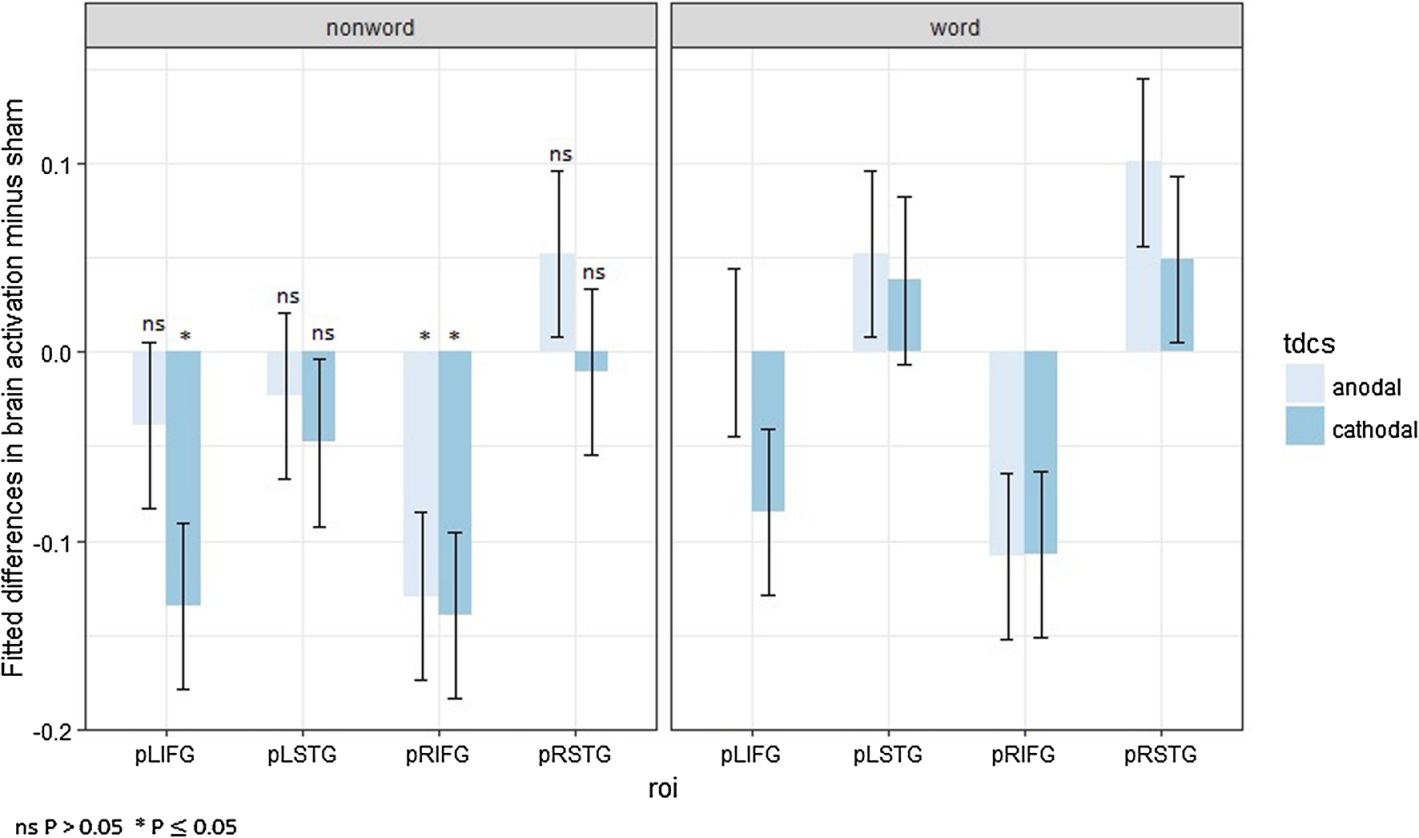
Fitted mean brain activation per ROI and stimulus type in lexical decision. The *x*-axis displays the ROIs. The *y*-axis displays the fitted mean brain activation. Error bars represent the contrast estimate ± the pooled standard error

Post hoc contrast analyses (Benjamini–Hochberg-corrected for multiple comparisons) were conducted, and some significant changes in activation were induced by both anodal tDCS and cathodal tDCS for the stimulus type nonword: cathodal tDCS on LIFG (*t*(19) = − 3.05, *p *= 0.046) and on RIFG (*t*(19) = − 3.17, *p *= 0.046), anodal tDCS on RIFG (*t*(19) = − 2.93, *p *= 0.046) (Fig. 6).

#### Connectivity analysis per stimulus type and tDCS condition

Partial correlation analyses were performed between the fitted mean brain activations of the target network ROIs by stimulus type and tDCS condition. Cathodal tDCS induced a larger number of significant correlations than anodal tDCS, as well as the stimulus type word induced a larger number of significant correlations than the stimulus type nonword. These occurred for the network connections LIFG/RIFG, LIFG/RSTG, RIFG/LSTG, RIFG/RSTG and LSTG/RSTG under cathodal tDCS with words, LIFG/RIFG, LSTG/RSTG and RIFG/LSTG under cathodal tDCS with nonwords and LSTG/RSTG under anodal tDCS with words. Anodal tDCS during nonwords induced no significant correlations between the target network ROIs (Tables 8, 9, 10, 11).

**Table 8:** Partial correlation analyses for fitted mean brain activations in words of lexical decision under anodal tDCS

	LIFG	LSTG	RIFG	RSTG
LIFG	1.00	− 0.26	0.27	0.34
LSTG	− 0.26	1.00	− 0.15	***0.85***
RIFG	0.27	− 0.15	1.00	0.26
RSTG	0.34	0.85	0.26	1.00

Pearson’s *r* for each pair of ROIs with significant correlations at *p* ≤ 0.05 marked in bold italic

**Table 9:** Partial correlation analyses for fitted mean brain activations in nonwords of lexical decision under anodal tDCS

	LIFG	LSTG	RIFG	RSTG
LIFG	1.00	0.10	0.31	0.17
LSTG	0.10	1.00	0.37	0.40
RIFG	0.31	0.37	1.00	0.10
RSTG	0.17	0.40	0.10	1.00

Pearson’s *r* for each pair of ROIs. No significant correlation at *p* ≤ 0.05 was observed

**Table 10:** Partial correlation analyses for fitted mean brain activations in words of lexical decision under cathodal tDCS

	LIFG	LSTG	RIFG	RSTG
LIFG	1.00	0.43	***0.87***	− ***0.58***
LSTG	0.43	1.00	− ***0.61***	***0.65***
RIFG	0.87	− 0.61	1.00	***0.72***
RSTG	− 0.58	0.65	0.72	1.00

Pearson’s *r* for each pair of ROIs with significant correlations at *p* ≤ 0.05 marked in bold italic

**Table 11:** Partial correlation analyses for fitted mean brain activations in nonwords of lexical decision under cathodal tDCS

	LIFG	LSTG	RIFG	RSTG
LIFG	1.00	0.43	***0.71***	− 0.09
LSTG	0.43	1.00	− ***0.64***	***0.58***
RIFG	0.71	− 0.64	1.00	0.35
RSTG	− 0.09	0.58	0.35	1.00

Pearson’s *r* for each pair of ROIs with significant correlations at *p* ≤ 0.05 marked in bold italic

### Analysis of words and nonwords in word naming

#### Effects of stimulus type, tDCS and ROI on mean brain activation

A 2 × 3 × 4 (stimulus type × tDCS × ROI) linear mixed effect model was fitted to the mean parameter estimates of ROI activation data of words and nonwords in the word naming task data of the healthy young adult sample. No significant two-way interaction was observed: stimulus type × tDCS (*F*(1,288) = 0.01, *p *= 0.90, *η*_p_^2^ < 0.001), stimulus type × ROI (*F*(3,288) = 0.20, *p *= 0.90, *η*_p_^2^ = 0.002) and tDCS × ROI (*F*(3,288) = 0.18, *p *= 0.91, *η*_p_^2^ = 0.002). The main effects of stimulus type (*F*(1,288) = 0.14, *p* = 0.70, *η*_p_^2^ = 0.001), tDCS (*F*(1,288) = 0.15, *p* = 0.70, *η*_p_^2^ = 0.001) and ROI (*F*(3,288) = 0.73, *p* = 0.54, *η*_p_^2^ = 0.01) were also nonsignificant.

**Fig. 7:**
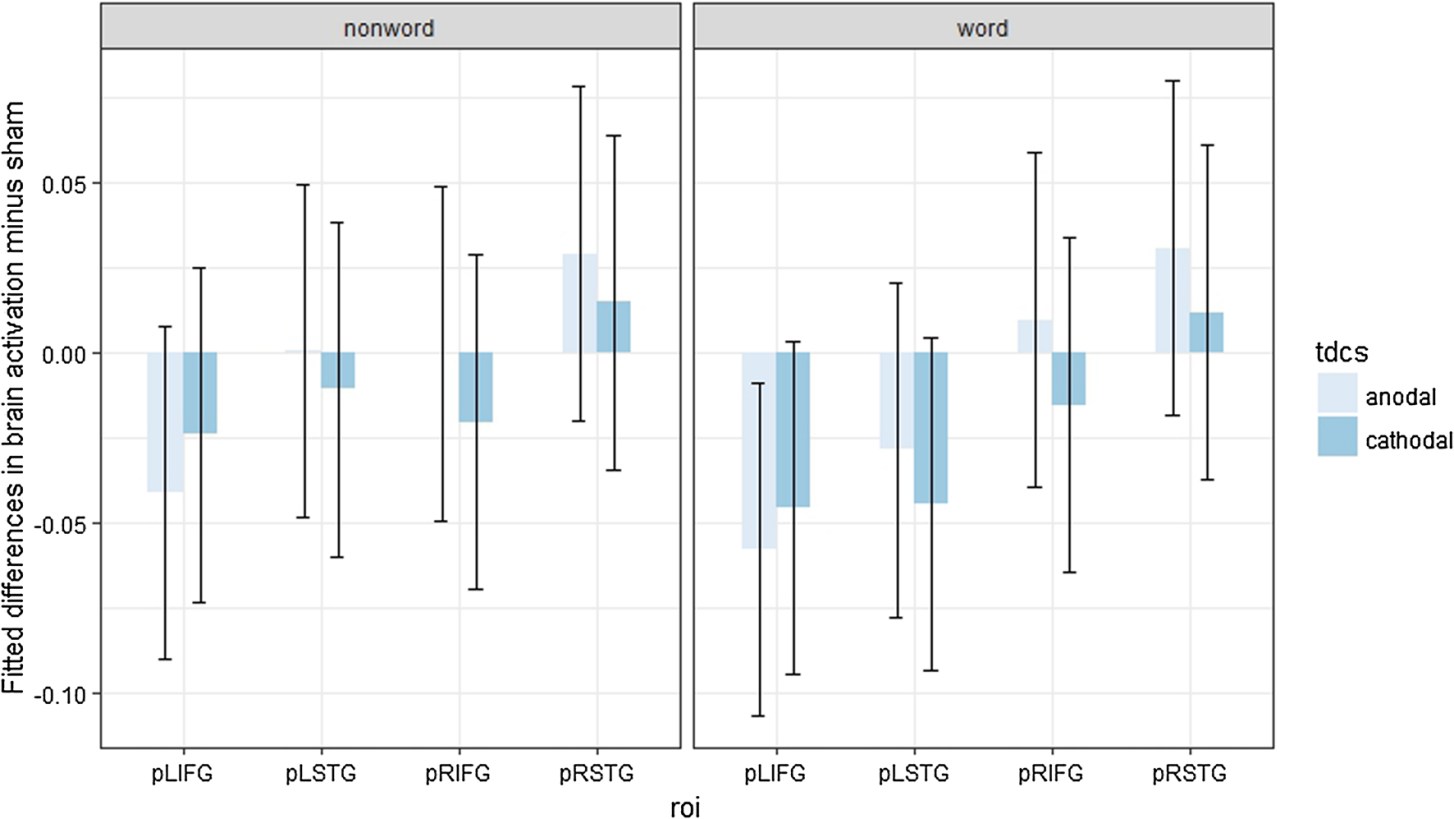
Fitted mean brain activation per ROI and stimulus type in word naming. The *x*-axis displays the ROIs. The *y*-axis displays the fitted mean brain activation. Error bars represent the contrast estimate ± the pooled standard error

Post hoc contrast analyses (Benjamini–Hochberg-corrected for multiple comparisons) were conducted, but no significant result was observed (Fig. 7).

#### Connectivity analysis per stimulus type and tDCS condition

Partial correlation analyses were performed between the fitted mean brain activations of the target network ROIs by stimulus type and tDCS. Under cathodal tDCS, the stimulus type word induced a larger number of significant correlations than the stimulus type nonwords: LIFG/RIFG, LSTG/RSTG and RIFG/RSTG for words and LIFG/RIFG for nonwords. Under anodal tDCS, both stimulus types induced significant correlations between the same pairs of nodes: LIFG/RIFG and LSTG/RSTG (Tables 12, 13, 14, 15).

**Table 12:** Partial correlation analyses for fitted mean brain activations in words of word naming under anodal tDCS

	LIFG	LSTG	RIFG	RSTG
LIFG	1.00	0.15	***0.74***	− 0.19
LSTG	0.15	1.00	0.23	***0.57***
RIFG	0.74	0.23	1.00	0.22
RSTG	− 0.19	0.57	0.22	1.00

Pearson’s *r* for each pair of ROIs with significant correlations at *p* ≤ 0.05 marked in bold italic

**Table 13:** Partial correlation analyses for fitted mean brain activations in nonwords of word naming under anodal tDCS

	LIFG	LSTG	RIFG	RSTG
LIFG	1.00	0.01	***0.68***	0.29
LSTG	0.01	1.00	0.04	***0.57***
RIFG	0.68	0.04	1.00	0.00
RSTG	0.29	0.57	0.00	1.00

Pearson’s *r* for each pair of ROIs with significant correlations at *p* ≤ 0.05 marked in bold italic

**Table 14:** Partial correlation analyses for fitted mean brain activations in words of word naming under cathodal tDCS

	LIFG	LSTG	RIFG	RSTG
LIFG	1.00	0.44	***0.71***	− 0.37
LSTG	0.44	1.00	− 0.08	***0.59***
RIFG	0.71	− 0.08	1.00	***0.48***
RSTG	− 0.37	0.59	0.48	1.00

Pearson’s *r* for each pair of ROIs with significant correlations at *p* ≤ 0.05 marked in bold italic

**Table 15:** Partial correlation analyses for fitted mean brain activations in nonwords of word naming under cathodal tDCS

	LIFG	LSTG	RIFG	RSTG
LIFG	1.00	− 0.05	***0.61***	− 0.03
LSTG	− 0.05	1.00	0.18	0.40
RIFG	0.61	0.18	1.00	0.28
RSTG	− 0.03	0.40	0.28	1.00

Pearson’s *r* for each pair of ROIs with significant correlations at *p* ≤ 0.05 marked in bold italic

## Discussion

In this study, we investigated whether the task load modulation of tDCS effects previously observed in behaviour (Rodrigues de Almeida et al. 2019), induced by varying motor participation in phonological processing across language tasks, could also be observed through differential patterns of functional network connectivity. We targeted the LIFG with tDCS and investigated with simultaneous fMRI, the network of interest underlying phonological processing, which consisted of the LIFG, RIFG, LSTG and RSTG nodes. Motor participation in phonological processing was assumed to translate into increasing task load for the LIFG across the speech perception to speech production range, which should in turn translate into different patterns of functional network connectivity during tDCS.

A categorical perception, a lexical decision and a word naming task were chosen to represent the speech perception to speech production range. Speech perception tasks were assumed to pose low task load on the LIFG (Lee et al. 2012; Liebenthal et al. 2013), while speech production tasks were assumed to pose high task load on the LIFG (Amunts et al. 1999; Eickhoff et al. 2009; Indefrey 2011; Liakakis et al. 2011). Words and nonwords were also tested in this study, because they were assumed to parallel the endpoints of the speech perception to speech production range of tasks, with words depending less on the LIFG than nonwords (Binder et al. 2003; Fiebach et al. 2002; Forster and Chambers 1973; Heim et al. 2005; Levy et al. 2009; Marshall and Newcombe 1973; Nosarti et al. 2010; Patterson and Shewell 1987; Xiao et al. 2005). To accomplish the aim of this study, outcomes of tDCS were analysed under the “multi-node framework” (Rodrigues de Almeida et al. 2019), which considers the impact of task load and network structures underlying cognitive functions on the outcomes of neurostimulation.

**Fig. 8:**
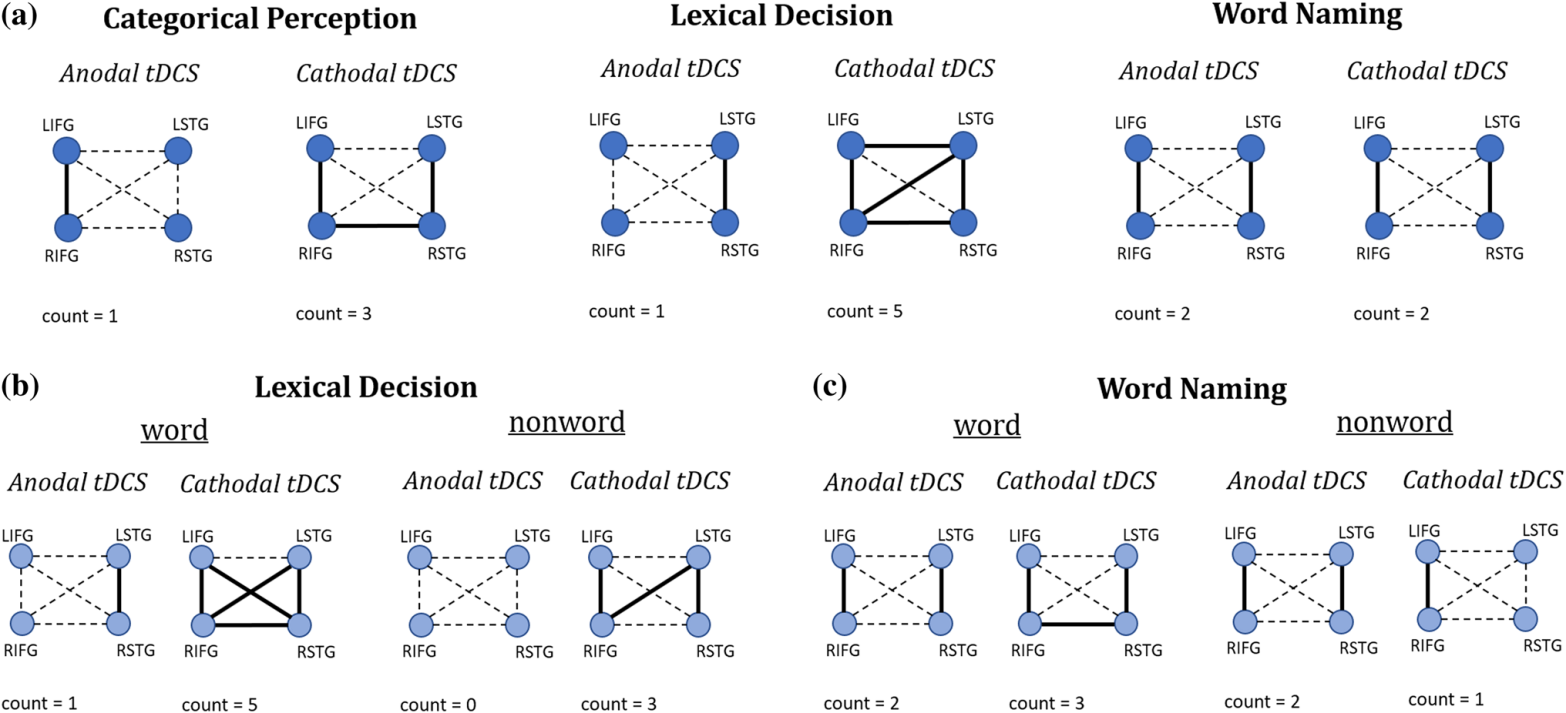
Summary of results per task, stimulus type and tDCS condition. Significant network connections are represented by full lines

Because tDCS effects positively relate to the task-induced level of neuronal engagement of the target (Bikson and Rahman 2013; Nozari et al. 2014; Pope et al. 2015), outcomes of stimulation over the LIFG in the current study were expected to differ across the speech perception to speech production range of tasks used. Excitation (under anodal tDCS) and inhibition (under cathodal tDCS) of the target should increase positively with task load (which increases from speech perception to speech production for the LIFG). Thus, cathodal tDCS should induce an amount of significant network connections negatively related to task load, indicating the amount of compensation available from other network nodes. Anodal tDCS was not expected to trigger compensation; therefore, any network activity induced should be comparatively smaller or null. ROI analyses (Fig. 8) revealed that task load and the network structure underlying cognitive functions are important factors that shape tDCS effects on network activity. In particular, task load across the speech perception to speech production range seems to play a role in the amount of network activity generated to compensate cathodal tDCS-induced downregulation of the target. Our measure of the number of significant connections arising for each tDCS/task condition relative to sham (Fig. 8) is believed to capture the global pattern of change in network activity that effectively took place in each condition. The specific identity of significant connections identified was additionally discussed or interpreted as appropriate.

Overall, present findings matched our predictions. For example, task load modulation of tDCS effects could in general be inferred from outcomes of cathodal tDCS. As predicted for this tDCS condition, tasks placed towards the speech perception end of the range (i.e. categorical perception and lexical decision) induced more network activity (as measured by the number of significant network connections) than the task on the speech production end (i.e. word naming). This was expected because, according to our model, when the target is downregulated in conditions of low task load it has more room for compensation than in conditions of high task load. Since tasks of speech perception are assumed to have less motor participation in phonological processing than tasks of speech production (Chang et al. 2010; Leonard and Chang 2014; Amunts et al. 1999; Eickhoff et al. 2009; Indefrey 2011; Liakakis et al. 2011), our tasks of speech perception were assumed to pose lower task load to the LIFG than our task of speech production. Therefore, our results are consistent with our model of functional connectivity in response to tDCS modulated by task load.

However, under the assumption that lower task load conditions generate larger amounts of compensation, our results would rate lexical decision, not categorical perception, as the task with the lowest task load for the LIFG, as it showed the highest number of significant connections during tDCS. However, in our previous study (Rodrigues de Almeida et al. 2019), no evidence of stronger compensation for lexical decision than for categorical perception was found, as cathodal stimulation produced a null result for the former task, and a statistically significant facilitation for the latter task. It may be that both tasks share similar processes in the perception end of the range to prevent differences in task load from being detected between them. Our results for the word and nonword stimuli matched predictions more accurately in both lexical decision and word naming tasks. Words, which were assumed to parallel the speech perception end of the range, triggered more network activity than nonwords, considered to parallel the speech production end of the range.

Anodal tDCS also induced some network activity consistent with work reported elsewhere (Baxter et al. 2017; Holland et al. 2016; Meinzer et al. 2012). However, no particular pattern regarding task load across the speech perception to speech production range was observed, suggesting that anodal tDCS effects on functional connectivity may be driven by factors other than task load. One idea from the literature is that anodal tDCS would have a general role in rendering network processing more efficient, as appropriate for each task (Meinzer et al. 2012). For example, Meinzer et al. (2013) improved performance of the elderly in an overt semantic word generation task by reducing their prefrontal hyperactivity with anodal tDCS. The authors targeted the left ventral inferior frontal gyrus with the stimulation, but observed with simultaneous fMRI that prefrontal hyperactivity was reduced bilaterally. It seems that in our study anodal tDCS similarly reduced prefrontal activity for the lexical decision task, as the only significant connection induced by the direct current was that between the temporal nodes. In contrast, a strong frontal connection might be crucial to handle task demands in categorical perception (Blumstein et al. 2005; Braber et al. 2005; Moineau et al. 2005), as the single significant connection between the LIFG and the RIFG found here for this task suggests. Of note, our results also showed, as predicted, that anodal tDCS induces less network response than cathodal tDCS, which is in line with the finding that anodal tDCS increases processing efficiency by reducing processing effort (Fiori et al. 2018; Holland et al. 2011; Meinzer et al. 2013).

Significant network connections involving right hemispheric nodes were induced by both cathodal and anodal tDCS in our study. This indicates that there was no particular preference for neural activity on the left hemisphere to accomplish phonological processing tasks, as it would have been expected if compensations were resolved within the putative pathway [namely: the dorsal pathway for phonological processing (Liebenthal et al. 2013; Saur et al. 2008)] subserving this function (Hartwigsen et al. 2012). On the one hand, this could mean that phonological processing, typically lateralised to the left hemisphere, may have a somewhat bilateral distribution in the brain (Hickok 2009; Liebenthal et al. 2013; Okada and Hickok 2006a, b). On the other hand, resorting to the right hemisphere may be a brain strategy used to deal with an increased demand to solve tasks that would otherwise rely predominantly on the left hemisphere. Findings reported elsewhere support this claim, showing that language processing demands may increase due to language impairment, age-associated cognitive decline, as well as to task difficulty and inhibitory brain stimulation (Dominguez et al. 2014; Gur et al. 2000; Hartwigsen et al. 2013; Meinzer et al. 2013; Vitali et al. 2007; Waldie et al. 2013). Of these reasons, task difficulty seems the best candidate to explain our findings, as it applies to all task conditions in our study and should impact outcomes regardless of tDCS polarity. In our work, all tasks were deliberately challenging in order to ensure sufficient neuronal engagement of the target during stimulation. Future work where task difficulty is manipulated may help to shed light on the role of this factor in the use of a right hemispheric strategy in phonological processing.

Contribution of a right hemispheric language strategy unfolds on a debate as to whether it is an expression of adaptive or maladaptive plasticity. Findings from the brain stimulation literature may shed light on this debate. For example, work by Hartwigsen et al. (2013) suggests that the right hemisphere strategy seems adaptive. In their study, the authors applied c-TBS to the LIFG of healthy participants during tasks involving the repetition of words and nonwords. A network response involving the right homologous region was observed, accompanied by improved performance. They suggested that the right hemispheric response, induced by the transient downregulation of the left hemisphere, parallels the brain reorganisation seen in acute or subacute stages of left-hemispheric post-stroke aphasia, where a right hemispheric strategy appears satisfactory (Saur et al. 2006). However, imaging work during language and nonlinguistic perceptual processing in healthy participants suggests that the increased right hemispheric activity for language in left-hemispheric stroke aphasia may actually reflect a compensatory increase in attention required to perform nonlinguistic aspects of language tasks (Baumgaertner et al. 2013). If so, this could be seen as a maladaptive *language* strategy, despite the satisfactory performance during language tasks when a right hemispheric response is present.

Other brain stimulation studies, including research with healthy participants (e.g. Costanzo et al. 2016a, b; Dominguez et al. 2014; Smirni et al. 2017; Torres et al. 2013), suggest that resorting to the right hemisphere to solve language tasks when left-hemispheric regions are able to perform the function is a maladaptive or suboptimal strategy and that enhancing activity in the left hemisphere is likely to induce satisfactory results. Dominguez et al. (2014), for example, enhanced performance in one participant with aphasia during phonological tasks by simultaneously applying cathodal stimulation to the RIFG, to reduce its hyperactivity, and anodal stimulation to the LIFG, to enhance its activity. Similarly, Costanzo et al. (2016a, b) showed that simultaneous tDCS with anodal stimulation over a left temporoparietal target and cathodal stimulation over its right homologous region, improved reading performance in participants with dyslexia, while reversing the stimulation polarity for the same targets hindered performance (Costanzo et al. 2016b). Similarly, Smirni et al. (2017) investigated phonemic fluency in healthy participants with rTMS and found decreased performance when inhibitory stimulation was applied to the LIFG, but increased performance when applied to the RIFG. The authors attributed task improvements to compensatory communication between the RIFG and the LIFG, supported by the LIFG. Present results alone cannot decipher whether the right hemispheric strategy observed in the target network for phonological processing expressed adaptive or maladaptive plasticity. Corresponding facilitatory effects on behaviour have been reported by Rodrigues de Almeida et al. (2019), but it remains to be seen whether a left-hemispheric strategy would have produced larger facilitation. Further behavioural and neuroimaging research targeting the RIFG with tDCS should be conducted to allow comparisons.

Finally, the current study also support a connectome approach to studying functional connectivity, whereby the neural connectivity profile, rather than localised brain activations, is responsible for the observed behaviour. We advocate that task load is a modulator of connectivity patterns. In our study, we demonstrated that task nature, which exert different loads for a given target of stimulation (i.e. the LIFG), is a modulator of connectivity responses during phonological processing. Similar language studies (Fuertinger et al. 2015) also demonstrate modulation by task nature. In short, the Fuertinger et al. (2015) chosen tasks represented an increasing hierarchy of difficulty in the speech production chain from baseline (rest) to production of meaningless syllables to production of meaningful sentences. Nonlinguistic control conditions (auditory perception and a motor task) were also tested. Using graph theory analyses, the authors identified a core hub network in the primary sensorimotor and parietal areas for all conditions, with the left posterior primary motor cortex especially involved in speech organisation. This hub network was involved with different functional domains across the networks subserving the different tasks used. Task nature modulated the level of participation of each slave network, which changed the topological configuration of the hub network.

We highlight that our work provided valuable evidence for task load modulation of tDCS effects on functional network connectivity as analysed with partial correlations, which is thought to be a reasonable standard technique to investigate functional connectivity when prior information on the temporal dynamics between nodes is not available for investigating causal relationships (Marrelec et al. 2006). But in order to further obtain information on the direction (forward or backward) and type of connections (excitatory or inhibitory) between nodes, future work that analyses the target network for phonological processing with effective connectivity is encouraged.

## Conclusions

In this study, we showed, with simultaneous tDCS and fMRI, that functional network connectivity patterns can be informative with regard to motor participation in phonological processing. As expected, motor participation was modulated by task nature across the speech perception to speech production range. Specifically, functional connectivity observed under cathodal tDCS of the LIFG seemed to follow a pattern related to task load, which allowed inferences about motor participation across the tasks to be made. This study, therefore, provided evidence that task load modulation of tDCS effects can be inferred from functional network connectivity patterns. Our results are consistent with those of our previous behavioural study (Rodrigues de Almeida et al. 2019), which together support key roles for “task load” and “network structure underlying cognitive functions” for the outcomes of tDCS (as advocated by the “multi-node framework”). Regarding anodal tDCS, the network activity observed is in line with previous findings, which associate anodal tDCS with a role in increasing network processing efficiency in a tailored fashion to the task at hand. Finally, this research helped to understand the patterns of functional network connectivity underlying motor participation in phonological processing, as well as the corresponding tDCS effects, in the healthy brain. Our findings help unravel functional network connectivity patterns resulting from phonological processing impairment, such as in aphasia or dyslexia, and to inform interventions with tDCS.
